# An efficient ANFIS-EEBAT approach to estimate effort of Scrum projects

**DOI:** 10.1038/s41598-022-11565-2

**Published:** 2022-05-13

**Authors:** Mohit Arora, Sahil Verma, Kavita, Marcin Wozniak, Jana Shafi, Muhammad Fazal Ijaz

**Affiliations:** 1 School of Computer Science and Engineering, Lovely Professional University, Phagwara, 144411 India; 2 Department of Computer Science and Engineering, Chandigarh University, Mohali, 140413 India; 3 Faculty of Applied Mathematics, Silesian University of Technology, 44-100 Gliwice, Poland; 4 Department of Computer Science, College of Arts and Science, Prince Sattam Bin Abdul Aziz University, Wadi Ad-Dawasir, 11991 Saudi Arabia; 5 Department of Intelligent Mechatronics Engineering, Sejong University, Seoul, 05006 Korea

**Keywords:** Mathematics and computing, Software

## Abstract

Software effort estimation is a significant part of software development and project management. The accuracy of effort estimation and scheduling results determines whether a project succeeds or fails. Many studies have focused on improving the accuracy of predicted results, yet accurate estimation of effort has proven to be a challenging task for researchers and practitioners, particularly when it comes to projects that use agile approaches. This work investigates the application of the adaptive neuro-fuzzy inference system (ANFIS) along with the novel Energy-Efficient BAT (EEBAT) technique for effort prediction in the Scrum environment. The proposed ANFIS-EEBAT approach is evaluated using real agile datasets. It provides the best results in all the evaluation criteria used. The proposed approach is also statistically validated using nonparametric tests, and it is found that ANFIS-EEBAT worked best as compared to various state-of-the-art meta-heuristic and machine learning (ML) algorithms such as fireworks, ant lion optimizer (ALO), bat, particle swarm optimization (PSO), and genetic algorithm (GA).

## Introduction

Estimating effort is a crucial component of software project management, especially when it comes to planning and monitoring a project. In software projects, cost and schedule overruns are recurring concerns. According to a study undertaken by Mckinsey and the University of Oxford on 5400 large-scale IT projects, large software projects run 66 percent over budget and 33 percent overtime on average^1^. As evident by Standish group chaos manifesto^2^, approx. 43 percent of the software projects entered crises as a result of wrong predictions of effort and its associated costs. Researchers explored and applied estimation types, techniques, and tools ranging from traditional to machine learning estimation for various agile methodologies. Software projects are complex and inherently uncertain which can be handled well by adaptive models. As per the systematic literature review published by Arora et.al^3^. IT managers working in Agile projects rely on traditional estimation techniques like planning poker, expert judgment, etc. which is suffered from individual bias. Our proposed approach has been inspired by the principles of adaptive networks and neuro-fuzzy to assist managers in deciding appropriate resources for the projects. The main ingredient of the proposed model is a hybrid neuro-fuzzy inference engine tuned by a novel EEBAT algorithm. Scrum project data has been seeded into the knowledge base to demonstrate the efficacy of the system. IT stakeholders are using issue tracking systems like JIRA^4^, which provides a holistic ecosystem to manage, integrate and collaborate end-to-end IT services but does not support Machine Learning assisted estimation. This paper makes an attempt to fill this void by not only narrowing down the actual-estimated effort gap but also producing results within the stipulated time and space constraints. The remaining work has been bifurcated as follows, “Related work” discusses related work, “Energy Efficient BAT (EEBAT) approach” describes Energy Efficient BAT approach, “Scrum effort estimation using ANFIS-EEBAT approach” discusses scrum estimation using ANFIS-EEBAT approach, “Experimental results and discussion” describes the experimental results and discussions, “Statistical validations” discusses various statistical tests to prove the effectiveness of the proposed model, “Threat to validity” discusses the threat to validity, and “Concluding remarks” discusses the concluding remarks.

## Related work

It can be inferred from the underlying literature that effort estimation has been the most targeted area of research in the software engineering domain because of its unquenchable need in IT and its associated industries. Process model transitions can be seen in the literature from waterfall to agile, thereby the traditional approaches of estimation like empirical, Delphi-Cost, etc., are not much suitable for estimation in the later^5^. Researchers have used machine learning techniques to bridge the gap of actual and estimated effort in agile inspired software and recorded a significant improvement. Agile aims to respond to changes positively thus soft computing techniques done justice by satisfying these inherent characteristics and provides reliable estimating. In a pool of wide variety of ML techniques, neuro-fuzzy frameworks^6^ assists well in establishing complex relationships between various people, process, and project attributes. The uncertainty of requirements and less available historical data makes training difficult and predictions vague. ML techniques have been used in conjuncture with a wide variety of optimizations^7^ like quality weighting in the analogy-based estimation, attribute weighting, tuning Artificial Neural Networks (ANN) adjustment (weight and bias), ANFIS adjustment, and variables positioning. Many prominent authors have reviewed and compared various regression-based and empirical techniques and found inferences wherein the former is outperforming the later with a significant margin. The study is not limited to few factors affecting estimation; instead, an exponential expansion can be seen vis-à-vis an increase in complexity of software projects. In some scenarios, conflicting outcomes have also been recorded in the literature irrespective of underlying process models. The estimation accuracy is changing with different datasets^8^ and/or scenarios^9^ using the same machine learning model. Authors are having conflicting interests’ w.r.t regression and other machine learning model comparisons. ANN and Case-Based Reasoning (CBR) correlation analysis has been carried out in Ref.^8^ and found ANN outperforms CBR whereas in Ref.^10^ detailed the contrary outcome.

In agile state-of-the-art reports and majority of literary resources, IT stakeholders carry out story point estimation, using Analogy, Planning Poker (PP), Expert Judgment (EJ), etc. A very few ML techniques have been applied in the field of agile estimating; however, it is needed the most, because of requirements volatility. They have either applied alone or in blend with other machine learning or non-machine learning methods^11–13^. GA has been used with CBR, ANN, and Support Vector Regressor (SVR) for hyper parameter tuning. Fuzzy logic^8^, Decision Trees (DT), Bayesian Networks (BN) with reviews attempted in the field of effort and cost estimation^10,14,15^. In a recent study of agile effort estimation, Deep Belief Network—Ant Lion Optimizer (DBN-ALO)^16^ hybrid approach has outperformed DT, Random Forest (RF) but they are expensive to train as it has complex data models. Authors in Ref.^17^ have created ensemble of Analogy and Artificial Bee colony for software development effort estimation. Ensemble are evaluated in Ref.^18^ and outperforming solo’s. A hydrid system based on Firefly algorithm for predicting maintainability emphasize on the change and quality management is used in Ref.^19^. Based on the trends and recorded observations by researchers, ML assisted estimation related literature has been presented in Table 1.

**Table 1:** ML Techniques used in agile scrum.

ML techniques	Accuracy parameter (MMRE)	Dataset used	Outperformed
Fireworks algorithm optimized Neural network^20^	MMRE-0.0293	21 projects developed by six software companies presented in Zia’s^21^ work	TLBO, TLBABC, DABC, LM
Multiagent Techniques^22^	MMRE—0.1	12 Web projects	Delphi, PP
Mamdani fuzzy inference systems^23^	MMRE (sprint1)-0.28 MMRE (sprint2)-0.15 MMRE (sprint3)-0.09	Three sprints of real soft- are projects	Comparison with actual estimate
General Regression Neural Networks (GRNN), Probabilistic Neural Networks (PNN), GMDH Polynomial Neural Network (GMDHPNN), Cascade Correlation Neural Network (CCNN)^24^	GRNN MMRE—0.3581 PNN MMRE—1.5776 GMDHPNN MMRE—0.1563 CCNN MMRE—0.1486	21 agile projects	Regression (Zia’s work), PNN, GRNN, GMDHPNN
Stochastic Gradient Boosting (SGB), RF, DT^25^	SGB MMRE—0.1632 RF MMRE—0.2516 DT MMRE—0.3820	21 agile projects	SGB outperformed RF, DT
Bayesian Networks^26^	Accuracy—Above 90% for six data sets	160 tasks in real agile projects	Comparison with an actual estimate
Hybrid ABC-PSO algorithm^27^	MMRE—0.0569	21 agile projects	ABC, PSO, GRNN, PNN, GMDPNN, CCNN
Support Vector Machine (SVM), NB, KNN, DT^28^	SVM MMRE—0.50 NB MMRE—0.85 KNN MMRE—0.70 DT MMRE—0.98	699 issues of inventive software designers, 5607 issues from 8 open source projects	Comparison with an actual estimate
Naïve Bayes (NB)^29^	MMRE—2.044	10 teams in IBM rational team concert	None

All the techniques mentioned and discussed in this section are derived from general estimation approaches to demonstrate a trail of estimation trends.

### Energy Efficient BAT (EEBAT) approach

The underlying architecture of the proposed approach has been inspired by the universal estimator i.e., Adaptive Neuro-Fuzzy Inference System^30^. ANFIS, in its original form, proved variously valued and promising solutions for problems of heavyweight process models in context to software estimation. ANFIS has some inherent pros and cons, which makes it a little less efficient for estimating in an agile environment if applied as a standard. Some shortcoming of ANFIS includes high computational cost due to complex structure and gradient learning hence for large inputs it will be slow, type and no. of membership functions, location of a membership function, curse of dimensionality and trade-off between interpretability i.e. rules and accuracy. As Agility is injecting ‘change’, a de-facto ingredient in the reshaping the culture of software engineering, it becomes a mandate to optimized ANFIS hyper parameters to predict and adjust scrum projects effort during all prominent sprints. EEBAT has optimized the learning curve of the base ANFIS.

### Standard ANFIS architecture

**Figure 1:**
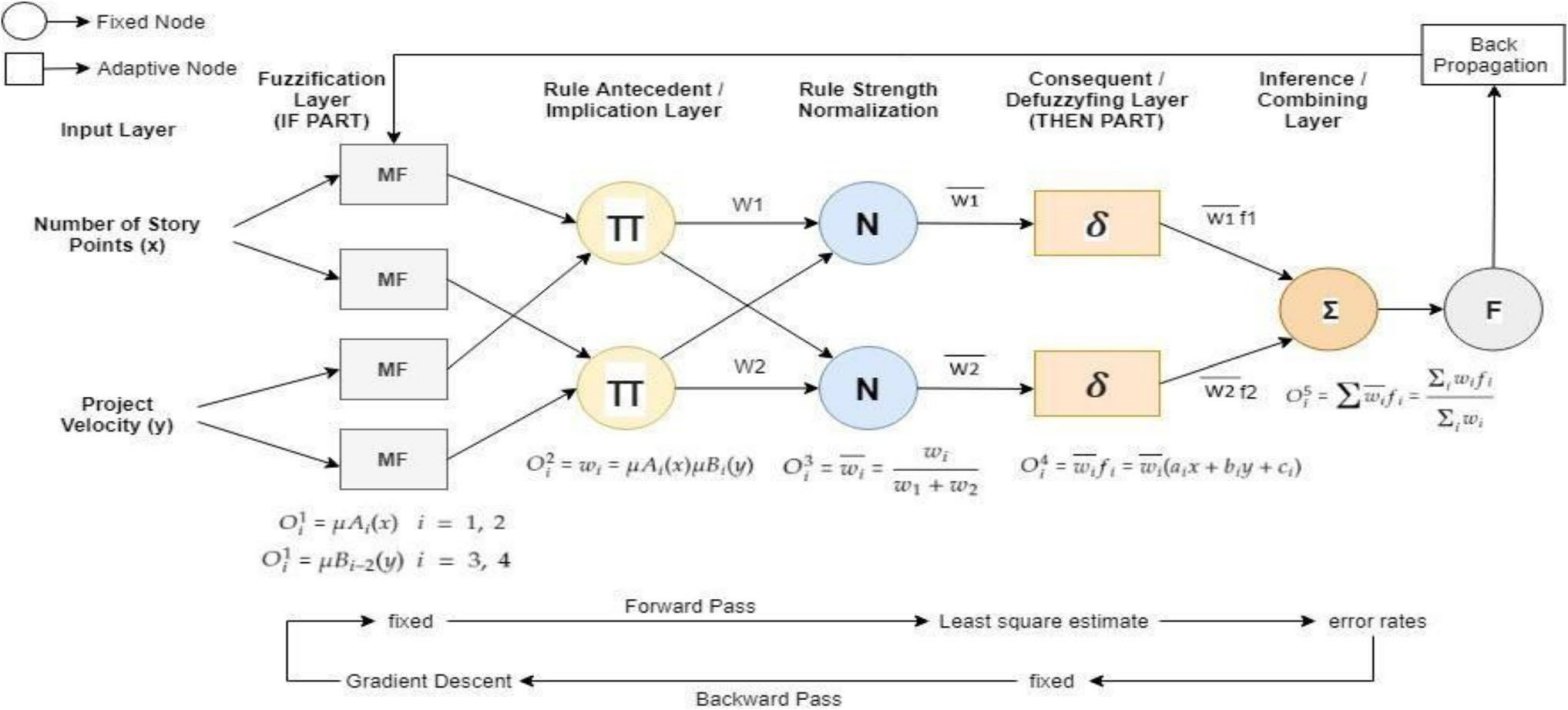
Standard ANFIS architecture. Number of Story Points and Project Velocity are given as inputs. These inputs have been derived from 21 projects dataset (dataset sample given in Table 2). ANFIS based exhaustive search MATLAB functionality has been used to decide the inputs. Different inputs (as feature pairs) tested against Actual Time. The feature pair with minimum error has been chosen in input layer.

Adaptive Neuro-Fuzzy Inference System, popularly known as a universal estimator and Takagi–Sugeno Fuzzy System makes use of potentials of both neural network and fuzzy logic in a package and is computationally more efficient than Mamdani, which mostly depends on the expert knowledge. The architecture of a standard ANFIS is given in Fig. 1 and has primarily five layers of perceptron’s or neurons in which perceptron's or neurons in the identical layer are alike and have similar functionalities as follows

- Fuzzifying layer: Each neuron is an adaptive node consisting of premise parameters.
- Implication layer: Neurons indicate the product of inputs.
- Normalizing layer: Each neuron is fixed.
- Defuzzyfing layer: Each neuron is also an adaptive one consisting of consequence parameters.
- Combining layer: It contains a single neuron that adds up all the inputs.

**Table 2:** Dataset sample.

Effort	Vi	D	V	Sprint size	Team size	Team salary	Actual time	Est time	Actual cost	Estimated cost	Time MRE	Cost MRE
156	4.2	0.687	2.7	10	22	230,000	63	58	1,200,000	1,023,207.14	7.93	14.73
202	3.7	0.701	2.5	10	21	260,000	92	81	1,600,000	1,680,663.89	11.95	5.04
173	4	0.878	3.3	10	22	250,000	56	52	1,000,000	992,269.51	7.14	0.77
331	4.5	0.886	3.8	10	22	300,000	86	87	2,100,000	2,002,767.22	1.16	4.63

In the sample dataset, there are 13 fields discussed below. Each row represents an agile Project. Projects names are not revealed in the referred dataset.

The Effort represents Number of Story Points.

V_i_ is Initial Team Velocity.

D is Deceleration, which affects the Team Velocity.

V is the Final adjusted team velocity, calculated by V = (V_i_)^D^.

Sprint Size is the size of a typical Sprint in a Scrum Project.

Team Size is the size of Scrum Team.

Team Salary is the salary of Scrum Team Members.

Actual Time field value represents Number of Days, for instance, in first row 156 Story Points (SP) took 63 days to complete Est. Time is the value of the estimated time calculated by Zia^21^ in his paper.

Actual Cost is the cost of Scrum Project.

Estimated Cost is the estimated calculated by Zia.

Time MRE is the value of Magnitude of Relative Error calculated by MRE(Time) = (Actual Time − Est. Time)/Actual time.

Cost MRE is calculated by MRE(Cost) = (Actual Cost − Estimated Cost)/Actual Cost.

### Energy efficient BAT approach

Standard BAT algorithm^31^ has certain inherent issues like failure to converge to global optima, multimodal optimization, poor exploration, slow rate of convergence, and no population diversity. To address these issues, various BAT variants have been introduced by researchers across the globe like Adaptive multi-swarm bat algorithm (AMBA)^32^, Bat with Mutation^33^, BATDNN^34^, Binary Bat algorithm, Differential Operator & Levy flights Bat^32^, Directed Artificial Bat Algorithm (DABA), Double- subpopulation Lévy flight bat algorithm (DLBA)^35^, Dynamic Virtual Bats Algorithm (DVBA)^36^, Improved Bat algorithm (cost estimation)^37^, Improved dynamic virtual bats algorithm with probabilistic selection^38^, Island multi populational parallel bat algorithm (IBA)^34^, Levy flight-based bat algorithm (LBA)^32^, LogisticBatDNN^33^, MeanBatDNN^33^, Modified Bat Algorithm (ANN)^33^, Modified Bat Algorithm (Stability Analysis)^39^, Multi-Objective bat algorithm (MOBA)^36^, Novel bat algorithm with multiple strategies coupling (mixBA)^40^, Piecewise-BatDNN^33^, shrink factor bat algorithm (SBA)^34^, Simplified Adaptive Bat based on frequency^41^, SinBatDNN^34^. Authors in Ref.^42^ endorsed the use of optimization techniques to reduce and determine effort of software projects. The list of inferences that have been deduced from these variants are: Handling trade-off between exploration and exploitation, Converging to global optima instead of being trapped in local minima, Flexibility in the integration of the bat variants in different models, Diversity factor to maintain the distinctness of population and Improvising the algorithm for multimodal functions.

In our proposed algorithm, we update the standard bat algorithm by considering a new parameter called Energy which will update the position and velocity of the bat based on its distance from the prey. We propose two new factors for the energy parameter—eagerness and magnitude of work, that dynamically get updated for controlling exploration and exploitation trade-off. It becomes exhaustive for a bat or pair of bats to search for its target or prey due to continuous echolocation (lack of cognitive ability), exploration (failure to converge), and exploitation (trapping in local optima). To address these concerns, EEBAT is proposed. The distinctive features of the proposed algorithm are—the energy parameter and memory capability. The Energy Parameter, E can be calculated using Eq. (1).

1 $$E={fitness}_{i}\times {mean(P}_{i}^{t}),$$

where *fitness*_*i*_, is the fitness of the current bat. The population diversity due to energy lets the bat intelligently assess its capability thus improving time complexity and convergence. The mean of the best positions is taken to find a convergence junction, as every bat in the population finds a different position for one value of the parameter. These positions are the best solutions as evident by the fitness value calculated so the collective energy of these deduced positions determines their optimality.

The memory capability of the bat, the population in standard bat has no history of the previous solutions encountered by the previous bats hence, novel solutions are left and premature convergence occurs. To solve this gap of the standard bat, the second improvement proposed is the introduction of memory capability. After every iteration, we store the position of bats in a special space called Memory Space (*MS*). This capability improves exploration as previously encountered solutions are prevented from being explored and exploited, hence improving the rate of convergence. This prevents the population from being trapped in local optima. It improves the time complexity of the algorithm.

### Scrum effort estimation using ANFIS-EEBAT approach

ANFIS provides increased learning, adapting, and non-linear abilities, as it makes use of combined advantages of Neuro and Fuzzy inference systems and thereby can be trained without an explicit empirical knowledge pool. Despite carrying strong estimation capabilities, ANFIS architecture needs parameter adjusting and tuning. The objective function of the ANFIS-EEBAT approach is to optimize parameters of ANFIS using an energy-efficient BAT algorithm. To begin with, the system needs its food to start estimating the effort of new projects. Our approach depends on the training of certain project parameters which will be primarily inserted in the knowledge base. However, the data needs to be understandable, so before training, it is being passed from the data preparation module. This section discusses our proposed algorithm ANFIS-EEBAT in context to effort estimation.

## Methodology

In “Methodology”, we have considered Six Software houses agile project data, as sample inputs, to begin with, mentioned in Table 2. The algorithm of the proposed methodology is presented in four broad categories given below.

### Dataset loading and feature selection

The dataset has been taken initially from six software houses which implemented agile-based projects and the following steps have been employed.

- Loading the agile project dataset.
- Perform a feature selection using an exhaustive search based on ANFIS.

### Data set partitioning and model selection

The transformed data will be split into training and testing sets.

- Partitioning of transformed data into training and testing sets in the ratio 80:20.
- Train the ANFIS-EEBAT model using training data.

### Testing part

In this part, model prediction on test data has been performed.

- Performing prediction using a trained model.
- Comparing prediction results with the original dataset.

### Performance evaluation

In this step, model performance will be evaluated through Squared Correlation Coefficient (R2), Root Mean Square Error (RMSE), Mean Absolute Error (MAE), Mean Absolute Percentage Error (MAPE), MMRE, and PRED. These performance evaluation metrices are defined as follows

- Squared correlation coefficient: It is used and defined to assess the efficacy of regression. It can be represented using the Eq. (2).
  2 $${R}^{2}\hspace{0.17em}=\hspace{0.17em}1-\frac{{\sum }_{i=1}^{N}(Actual\, Effort-Estimated \,Effort)^2}{{\sum }_{i=1}^{N}(Actual \,effort-Mean\left(Actual\, Effort\right))^2}$$
- Mean absolute percentage error: It determines absolute accuracy for different estimation models. The term absolute is considered as the assessment of the cost estimations from the actual recognized costs. MAPE can be calculated using the Eq. (3).
  3 $${MAPE}_{i}=\frac{1}{N}{\sum }_{i=1}^{N}|\frac{Actual\, Effort-Estimated\, Effort }{Actual \,Effort}|\times 100$$
  In this, the first summation is done for each estimated point, divided by the number of suitable points N.
- Prediction (PRED (x)): In mathematical definition, PRED(x) is mathematically determined as Eq. (4)
  4 $$PRED\left(x\right)\equiv \sum_{i=1}^{N}\left[{MRE}_{i}\le x\right]) | N>0$$

PRED(x) value is calculated using the Eq. (5).

5 $$PRED\left(x\right)=\frac{K}{N}.$$

Here, ‘N’ represents the total of projects and ‘K’ is the count of projects having MRE below or equal to x. The value of x can be either 0.25, 0.50. 0.75 or 1.0. If a common value of x is 0.50, then PRED (0.50) refers to the % of projects whose MRE is less than or equal to 50%. Measuring the accuracy of estimation in scrum is an essential activity and determines its superiority with self and others.

- Perform model comparison using various performance metrics.
- Compare the output of the above defined metrics

### Deducing optimal parameters from EEBAT

The proposed system after the default initialization process will undergo tuning of base fuzzy system parameters by EEBAT. The inherent training algorithm of ANFIS will be replaced by EEBAT. The parameters of base fuzzy system will be adjusted based on fitness/error function Mean Magnitude of Relative Error (MMRE) which should be low, as given in Eq. (6).

6 $$\mathrm{MMRE }=\frac{{\sum }_{i=1}^{N}\frac{|estimated-actual|}{actual}}{N}.$$

**Figure 2:**
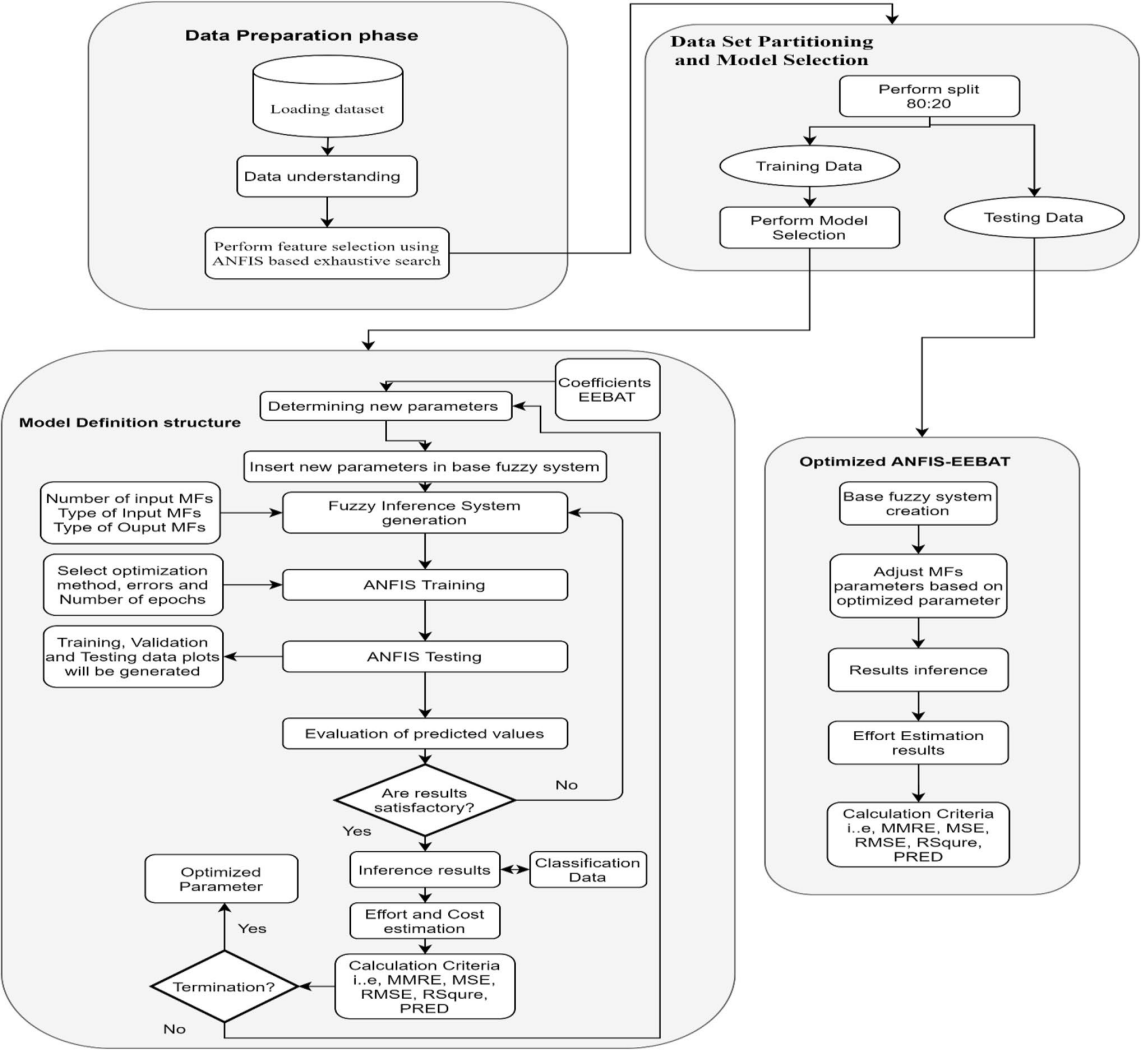
ANFIS-EEBAT for effort estimation. This figure gives a pictorial view of the detailed supposition stages of Scrum Projects Effort Estimation. EEBAT algorithm will determine new parameters for ANFIS. The training and testing of optimized ANFIS will be carried out. The results of estimated Effort are given in the upcoming section.

Here, N is the number of projects in the dataset *genfis* is used as a base fuzzy system with *fuzzy c-means* clustering to create rules and input MFs in the forward pass. EEBAT will minimize the error in the backward pass run. The detailed supposition stages of effort estimation are given in Fig. 2.

### Employing optimized parameters in ANFIS obtained by EEBAT

In this step, values of error metrics, e.g. MMRE will be observed. The optimized parameters obtained in the previous section will be initialized as default parameters of MFs of base fuzzy system.

## Experimental results and discussion

The accuracy achieved by the system depicts the efficacy of the proposed system. Many researchers have presented their hybrid approaches by incorporating meta-heuristic algorithms for parameter(s) optimization.

### Dataset sample

The dataset sample is given in Table 2. The dataset has been taken from Zia^21^.

### Renaming, identification and selection of features and labels

We have renamed few fields of Dataset and performed ANFIS based exhaustive search to find the best combination of fields which is chosen as inputs *aka* features and is matched against output *aka* label. This exhaustive search has been carried out in MATLAB. Fields named “Effort”, “V” and “Actual Time” from Table 2 is renamed to “No. of Story Points”, “Velocity” and “Actual Effort” respectively. Table 3 shows that our label “Actual Effort” is mostly affected by “No. of Story Points” and “Velocity” with minimum value of Train error i.e., 0.6504. The other pairs (No. of Story Points − Team Size) and (Velocity − Team Size) has not been selected as the value of the train error is more vis-à-vis chosen pair. This section assists IT managers in making better decisions of features selection.

**Table 3:** Feature analysis table.

Features	Train error
No. of story points, velocity	0.6504
No. of story points, team size	4.9212
Velocity, team size	15.7069

The least indispensable features selection minimizes complexity and produce software effort estimation results in less time^43^.

The deduced features and label after renaming is given in Table 4.

**Table 4:** Dataset features and labels.

Features		Labels
No. of story points	Velocity	Actual effort
156	2.7	63
202	2.5	92
173	3.3	56
331	3.8	86

### Expansion of dataset using k-means SMOTE

We have applied k-means based Synthetic Minority Over Sampling Technique (SMOTE) using Eq. (7), a data augmentation technique on Zia dataset, to generate synthetic values of features and labels. The purpose of this step is to address the issues of a modest amount of data for training and testing.

7 $$x^{\prime } = x + {\text{rand}}\left( {0,{ 1}} \right) \times |x - x_{k} |.$$

Here, *x* is the element of minority class set *A*, is the element of a set *A*_1_ which is calculated using k nearest neighbors of *x*, sampled at some rate *N*. The new dataset is labeled as ZKmS (Zia K-means SMOTE) and is being used in our ANFIS-EE- BAT model.

### Descriptive statistics of the dataset

The descriptive statistics of ZKmS has been given in Table 5. It includes count (number of projects in the dataset), mean, and standard deviation, minimum and maximum value of “No. of Story Points”, “Velocity” and “Actual Effort” in dataset.

**Table 5:** Descriptive statistics of the dataset.

Statistics	No. of story points	Velocity	Actual effort
Count	162.000000	162.000000	162.000000
Mean	159.648148	3.054938	54.333333
Std	72.914182	0.384328	23.046806
Min	62.000000	2.400000	21.000000
Max	339.000000	4.200000	112.000000

The statistics “Count” with value 162 signifies that ZKmS contains 162 projects data. “Mean” represents the average value of the fields. “Std” is the standard deviation which represents the difference of the field values from the Mean value. “Min” and “Max” show the minimum and maximum value respectively.

### Model selection

ANFIS-EEBAT has been applied to the features from the dataset as per the step given below.

### Data loading and generate fuzzy inference system

After we input features in the proposed ANFIS-EEBAT model, the antecedent layer creates the input MFs. The initial set of parameters for ANFIS and EEBAT are given in Table 6. The values of ANFIS parameters have been optimized using EEBAT.

**Table 6:** ANFIS and EEBAT parameters.

ANFIS parameters		EEBAT parameters	
Number of inputs	2	Population size	40
Number of outputs	1	Max iterations	100
Learning algorithm	EEBAT	Pulse rate	0.3
Number of inputs MFs	[4 4]	Loudness	0.9
Partitioning method	Fuzzy C-means	fmin	0
Input MF type	gaussmf	fmax	0.1
Output MF type	Linear	Alpha	0.9
Base fuzzy system	genfis3	Gamma	0.9
And method	Prod		
Or method	Probor		
Implication	Min		
Aggregation	Max		
Defuzzification	wtaver		
Maximum iterations	100		
Error tolerance	1e−5		

### Building ANFIS-EEBAT model structure

**Figure 3:**
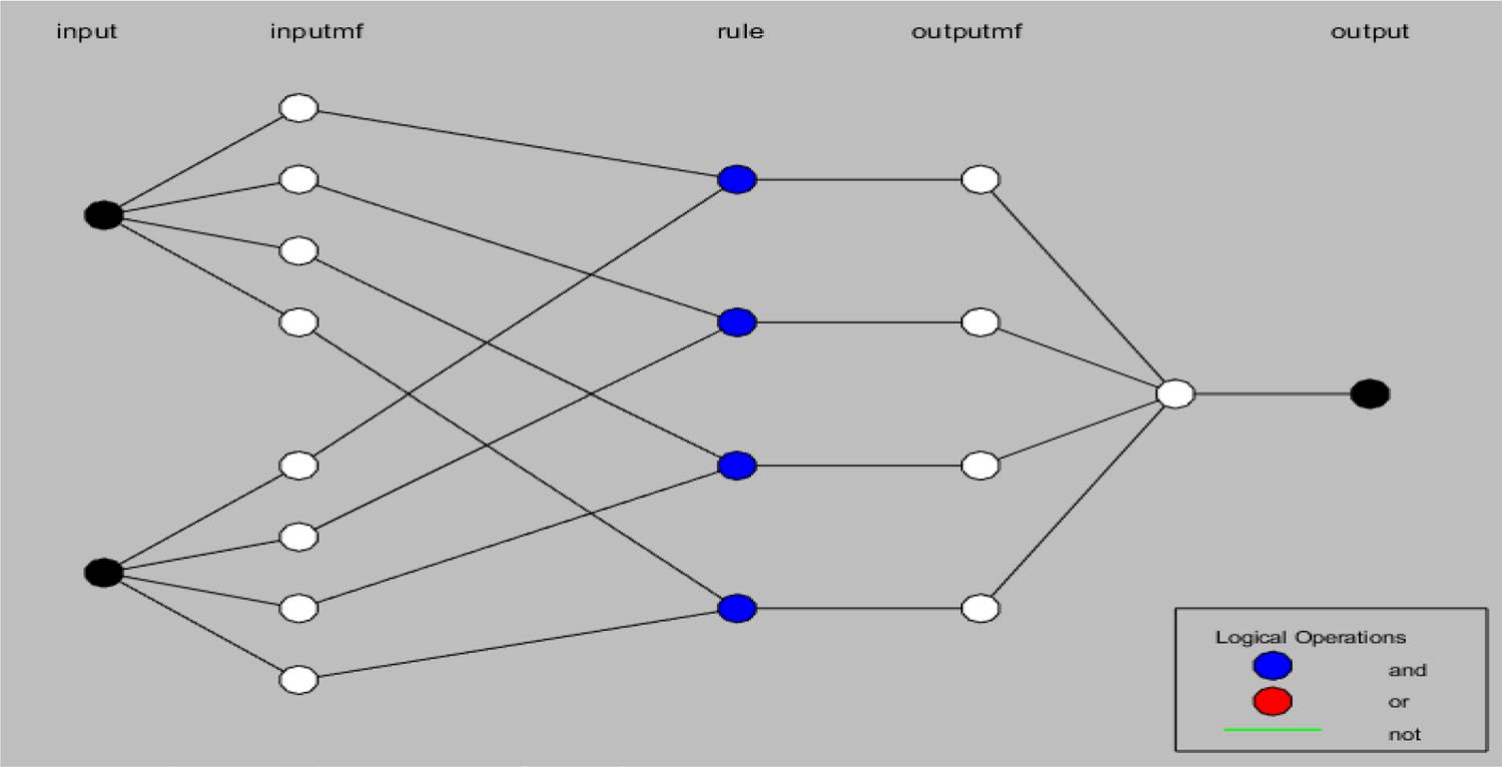
ANFIS-EEBAT structure. It contains five layers, as discussed in Fig. 1. There are two inputs, four pairs of input MFs, four set of rules, four output MFs and one output. The two inputs are “No. of Story Points” and “Velocity”. The output is “Estimated Effort”. The operations performed at different layers are synonymous with the description in Fig. 1. There are three basic logical operations, “and”, “or”, “not” depicted in the figure with three color codes “blue”, “red” and “green” respectively. The rules are created using logical “and” operation in our case. The logical “or” and “not” operations are unused.

After setting up the initial parameters, the proposed model’s structure is shown in Fig. 3.

The ANFIS and EEBAT parameters are explained in the Table 6. The Number of inputs is “2” which are “No. of Story Points” and “Velocity”. The Number of outputs is “1” which is “Actual Effort”. The learning algorithm is “EEBAT”. The value “4” in number of inputs MFs parameter signify that there exists 4 Gaussian MFs for each input with unique set of Gaussian parameters. “Fuzzy C-Means” partitioning method has been employed which is used to create base fuzzy inference sys- tem. The input MF is “gaussmf (Gaussian)” that represents our data in normal distribution and the output MF is “linear” which produces a singular value. The base fuzzy system is created using “genfis3” functionality of MATLAB. The “And” method signifies the product of weights of neuro-fuzzy system with the in- puts. The “Or” method utilizes “probor (probabilistic or)” which is the algebraic sum of the previous layers. The implication and aggregation are set to “min” and “max” respectively. “wtaver” i.e., weightage average is used for defuzzification. The training iterations aka epochs are set to 100 as after this value over fitting occurs. The iterations have been validated against several trials. The error tolerance is set to 1e−5. The initial BAT population is set to “40”. The maximum number of iterations is “100”. Pulse rate signifies optimal solution searching precision of the algorithm. The tuning parameters of ANFIS are the optimal solution. Loudness controls the speed of convergence of the algorithm. The value of fmin and fmax determines the range of frequency, which assists in global searching capability. Alpha and gamma are constants. The values for each parameter are obtained during several exhaustive trials.

### ANFIS-EEBAT MFs and rules view

**Figure 4:**
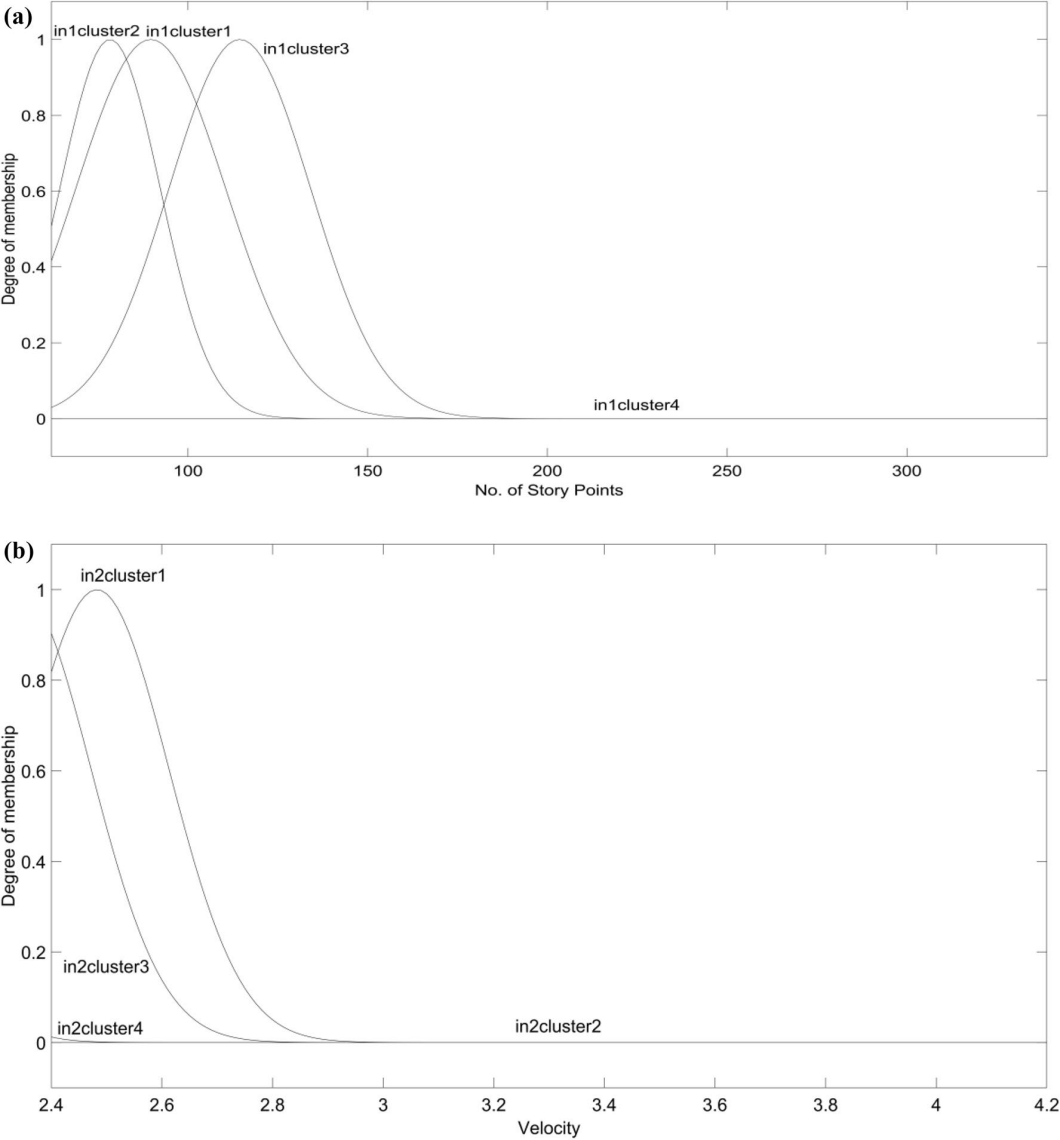
(**a**) Membership function for Input1 i.e., No. of Story Points. The x-axis and y-axis represent “No. of Story Points” and membership values of input1 respectively. The set of four unique MFs created are represented as curves (in1cluster1, in1cluster2, in1cluster3, in1cluster4) in the figure. (**b**) Membership function for Input2 i.e., Velocity. The x-axis and y-axis represent “Velocity” and membership values of input2 respectively. The set of four unique MFs created are represented as curves (in1cluster1, in1cluster2, in1cluster3, in1cluster4) in the figure. The curves are overlapped due to the minute values of “Velocity” which is ranging from 2.4 to 4.2.

**Figure 5:**
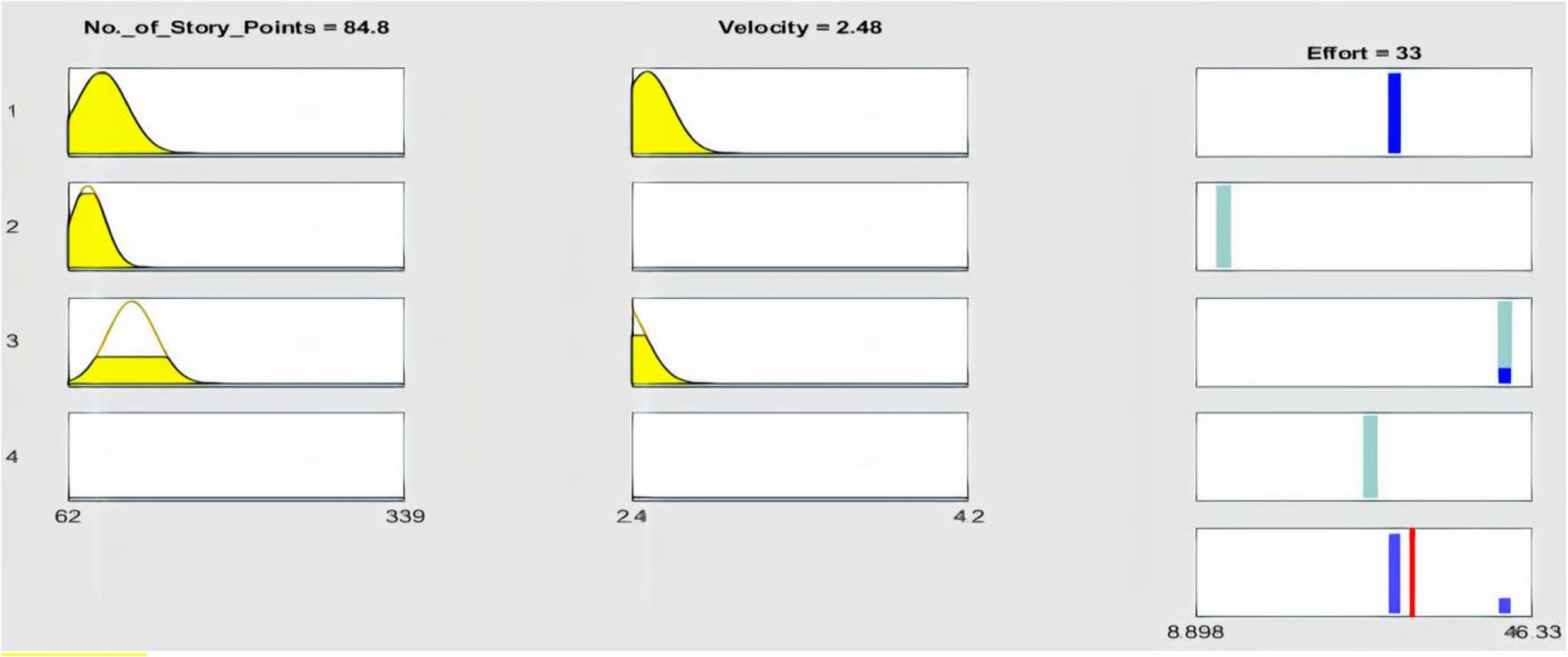
ANFIS-EEBAT rules view. Each row of plot represents a rule. There are a set of four rules based on input and output membership functions. The red line is a slider for selecting the input values. For instance, we have selected the value of “No. of Story Points” as 84.8 and “Velocity” as 2.48. The yellow color in the plots depicts how input variable is used in the rules. The blue color in the output membership function, “Effort”, signifies how output is utilized in the rules. The output of each rule is combined and defuzzified to create an aggregated output in the bottom-right plot. The estimated effort, 33 is the output and is shown by the red color line.

After the training and testing, membership function parameters are adjusted using EEBAT and can be seen in Fig. 4a,b. The rules for the same are shown in Fig. 5.

### ANFIS-EEBAT surface plot

**Figure 6:**
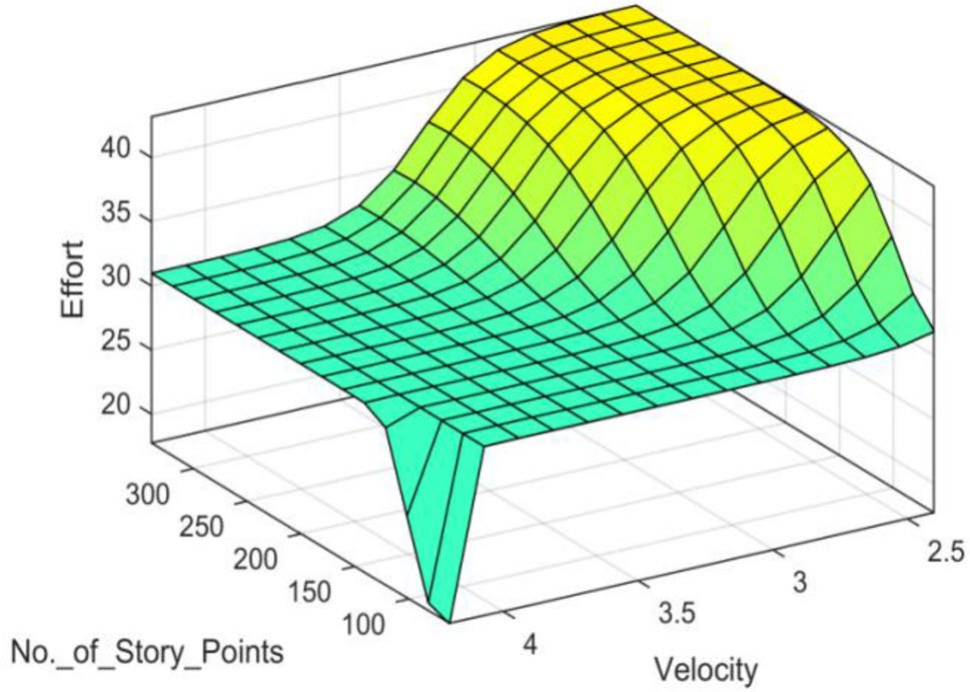
ANFIS-EEBAT surface plot.

The surface plot shown in Fig. 6 depicts the mapping of the features with the labels. It can be deduced from the surface plot that for our features, the output is linear, which is following the Takagi Sugeno type 3 Fuzzy Inference System (FIS).

### ANFIS-EEBAT performance evaluation

ANFIS-EEBAT model’s performance has been evaluated using various metrics such as Squared Correlation Coefficient (R^2^), Root Mean Square Error (RMSE), Mean Absolute Error (MAE), Mean Absolute Percentage Error (MAPE), MMRE, and PRED and is given in Table 7 for ZKmS and Zia datasets. ANFIS-EEBAT has also been compared with other state-of- the-art models on aforementioned datasets and summarized in Tables 8 and 9. Our approach is accurate to 98.47% and 99.93% on ZKmS and Zia datasets, respectively and will assist the IT industry stakeholders in getting accurate estimates of their respective projects. It also provides 100% estimation accuracy up to 2.4% for PRED. The RSquare value for the ANFIS-EEBAT is very high and can be seen in the Table 7 (0.98472 for ZKmS and 0.99934 for Zia datasets). As a result, there is a strong positive link between the story point, velocity, and the estimated work necessary to develop the software, with little changes in one causing considerable changes in the other.

**Table 7:** ANFIS-EEBAT performance metric evaluation.

Dataset	R^2^	RMSE	MAE	MAPE	MMRE	PRED (25%)	PRED (2.4%)
ZKmS	0.984723	2.827164	0.440483	0.971148	3.910372	100	100
Zia^17^	0.999349	0.74579	0.35558	1.019133	1.518311	100	100

**Table 8:** Results on ZKmS with other techniques.

Techniques	R^2^	RMSE	MAE	MAPE	MMRE	PRED (15%)
ANFIS	0.982857	3.042915	0.57697	0.896473	4.310884	100
ANFIS-GA	0.973329	4.269707	2.025023	3.525099	6.568641	96.67
ANFIS-PSO	0.977309	3.439497	0.366967	0.857856	4.498164	96.67
ANFIS-BAT	0.955252	4.972847	1.386903	2.748092	5.78877	86.67
Random forest	0.812542	9.935695	0.325292	1.394628	13.38908	66.67
SVR	0.294153	19.4183	3.678706	0.965226	20.50747	46.67
SGB	0.955736	4.759849	0.35478	0.825001	5.676487	93.33
ANFIS-EEBAT	0.98472	2.82716	0.44048	0.97115	3.91037	100

**Table 9:** Results on Zia with other techniques.

Techniques	R^2^	MAE	MMRE	PRED (25%)	PRED (2.4%)
ANFIS	0.982857	0.57697	4.310884	100	40
ANFIS-GA	0.973329	2.025023	6.568641	100	20
ANFIS-PSO	0.977309	0.366967	4.498164	100	40
ANFIS-BAT	0.955252	1.386903	5.78877	100	40
Zia regression^17^	Not available*	Not available	7.19	57.14	0
Fireworks algorithm^16^	0.9946	Not available	2.9339	Not available	Not available
DBN-ALO^13^	Not available	Not available	2.225	98.4321	Not available
ANFIS-EEBAT	0.99935	0.35558	1.518	100	100

**Figure 7:**
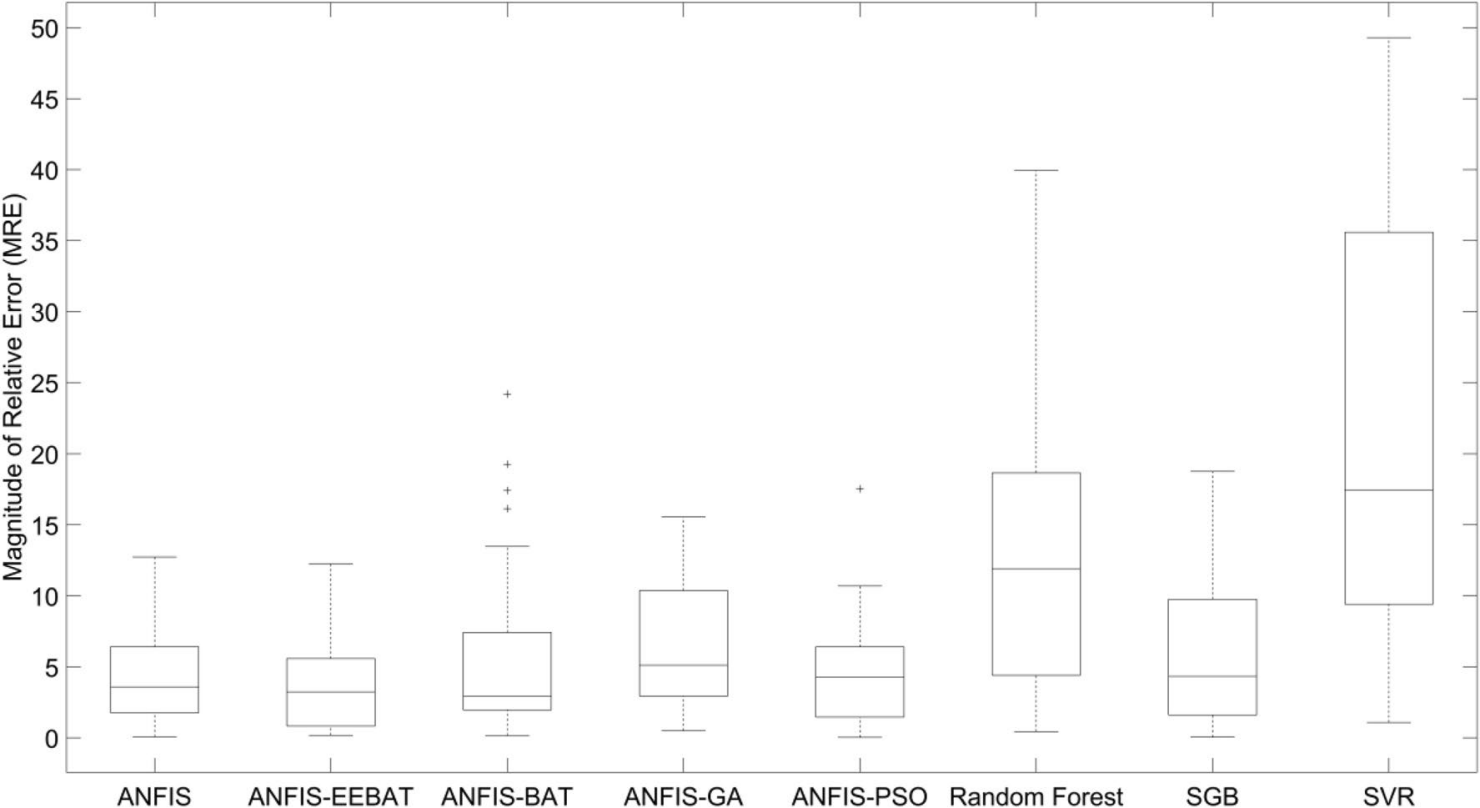
Box Plot of ANFIS-EEBAT with other models on ZKmS dataset. It can be inferred from the plot that ANFIS-EEBAT has the lowest value of the median.

*The data of performance metrics is not available in the referred research papers. This comparison has been performed on the real agile projects. It can be inferred that in spite of good accuracies by Fireworks optimized neural network and Deep Belief Network-Ant Lion Optimizer (DBN-ALO), a gap of actual and estimated effort is still present. This gap has been further narrowed down using ANFIS-EEBAT approach with a PRED of 100 close to 2.4%. Figure 7 depicts the Box Plot of ANFIS-EEBAT with other models.

The lowest MMRE and highest PRED (15%) signify the efficacy of ANFIS-EEBAT over other techniques. Various techniques are employed on ZKmS dataset for comparative analysis. Standard ANFIS uses hybrid (Backpropogation and Least Square Regression (LSE)) learning for training. In ANFIS-GA, ANFIS-PSO and ANFIS-BAT the default learning algorithm of ANFIS have been replaced by Genetic Algorithm (GA), Particle Swarm Optimization (PSO), and BAT, respectively. GA, PSO and BAT are well known nature inspired meta-heuristic algorithms. Their innate ability of finding optimal solutions provides valuable feedback in exploration and comparison. Random Forest is an ensemble learning method which performs mean prediction of singular trees for estimation. Radial Basis Function (RBF) kernel-based Support Vector Regressor (SVR) has been used. Stochastic Gradient Boosting (SGB) has also been employed for estimation. It is a well-known algorithm which inculcates randomness and variation in boosting which increases robustness in learning complex data.

## Statistical validations

**Figure 8:**
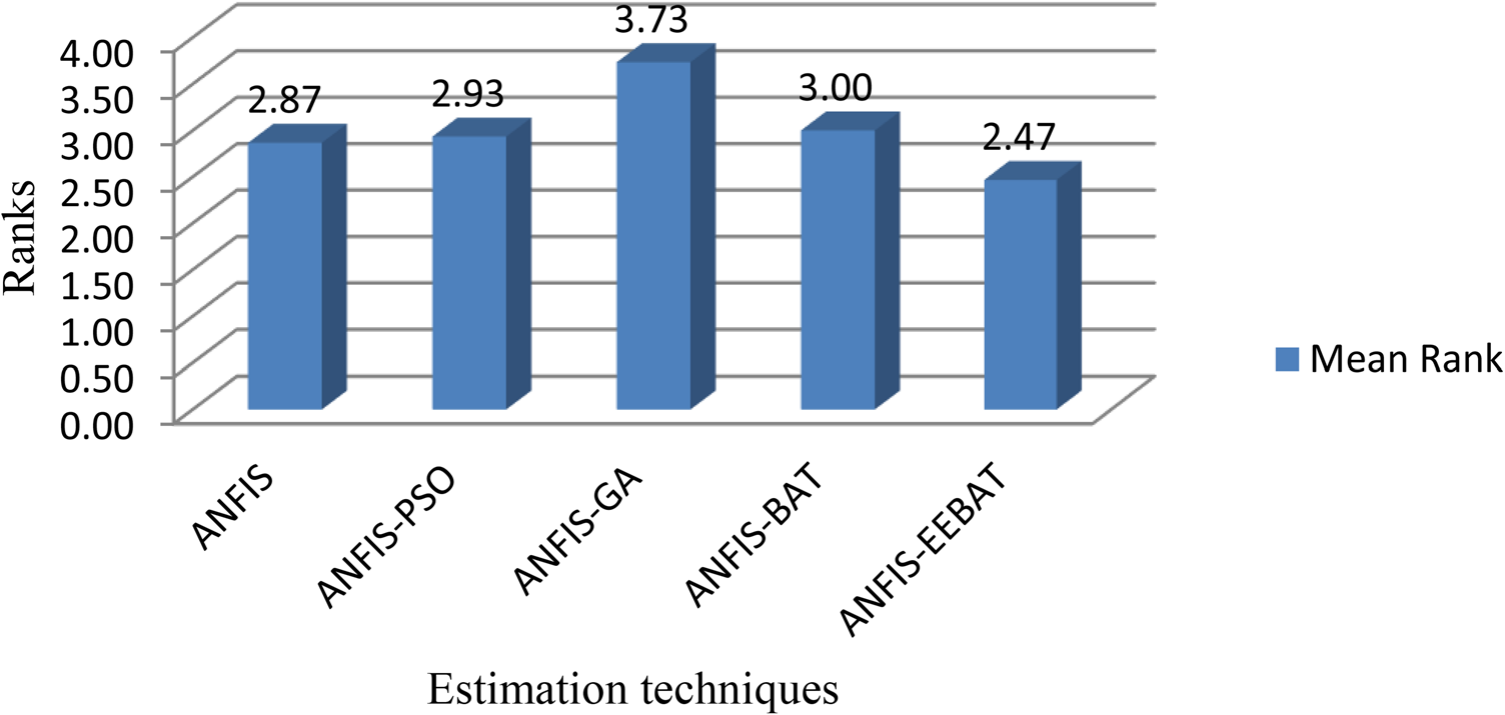
Friedman test rank comparison on ZKmS We have considered our null hypothesis that all the models are similar. The standard chi-square value for 4 degrees of freedom, df, and alpha = 0.05 is 9.488 and our chi-square value is 10.133, which rejects our null hypothesis. The value of Asymp. Sig. (0.038) is less than the significance value of alpha (0.05) which ascertains that models are dissimilar. The mean rank of ANFIS-EEBAT is 2.47 as compared to ANFIS (2.87), ANFIS-PSO (2.93), ANFIS-GA (3.73) and ANFIS-BAT (3.0).

Because the dataset in software effort estimating studies does not fit into any particular distribution, nonparametric tests are advised^44^. As per the nature of our data, non-parametric tests such as Friedman^45^ have been applied to the ZKmS dataset using SPSS. The average ranking of the models using this test is shown in Fig. 8. The test provides the lowest rank to the best technique.

## Threat to validity

The dataset has been generated using SMOTE (k-means) from the original agile data taken from six software houses and the proposed algorithm has been applied on both Zia and ZKmS to validate its efficiency. However, it can be validated on more datasets.

## Concluding remarks

Estimation is an indispensable requisite that assist project managers to take firm decisions and fulfilling client commitments. As per the current literature, during the start of any typical IT project, managers primarily depend upon the empirical estimation. Due to the complex nature of projects, estimation based on an educated guess does not yield fruitful results. Machine Learning assisted estimation, narrows down the gap of actual and estimated effort to a substantial level. We have attempted to bridge the aforementioned gap to a greater extent using the ANFIS-EEBAT approach. Our approach is making use of the three capabilities viz, neural networks, fuzzy, and novel BAT. The complexity of the proposed algorithm is managed by our novel energy equation and memory space concept. This work can be extended using other optimization algorithms like firefly, Sail Fish Optimizer.

## Data Availability

The data shall be made available on request.
